# Low Skeletal Muscle Radiodensity Predicts Response to CDK4/6 Inhibitors Plus Aromatase Inhibitors in Advanced Breast Cancer

**DOI:** 10.1002/jcsm.13666

**Published:** 2024-12-17

**Authors:** Hyunwook Kim, Seungjin Baek, Sookyeong Han, Gun Min Kim, Joohyuk Sohn, Yumie Rhee, Namki Hong, Min Hwan Kim

**Affiliations:** ^1^ Department of Internal Medicine, Division of Medical Oncology, Yonsei Cancer Center Yonsei University College of Medicine Seoul South Korea; ^2^ Department of Internal Medicine Yonsei University College of Medicine Seoul South Korea; ^3^ Endocrine Research Institute Severance Hospital Seoul South Korea; ^4^ Department of Internal Medicine, Endocrine Research Institute, Severance Hospital Yonsei University College of Medicine Seoul South Korea

**Keywords:** breast cancer, CDK4/6 inhibitors, CT‐derived body composition, low skeletal muscle radiodensity, muscle density

## Abstract

**Background:**

Recent evidence indicates that a dysregulated host metabolism influences treatment outcomes in patients with breast cancer. We investigated the association of computed tomography (CT)‐derived body composition indices with therapeutic responses in patients with hormone receptor‐positive, HER2‐negative advanced breast cancer (ABC) on endocrine plus CDK4/6 inhibitor (CDK4/6i) treatment.

**Methods:**

The study involved a retrospective cohort of patients with ABC at the Yonsei Cancer Center who received CDK4/6i and aromatase inhibitors as first‐line therapy between January 2017 and October 2020. Body composition parameters were estimated from the non‐enhanced CT images of the third lumbar spine by commercialized deep learning software. Patients with low skeletal muscle radiodensity (SMD) were defined as patients with SMD of low tertile (≤ 28.7 Hounsfield Units). The primary outcome was progression‐free survival (PFS).

**Results:**

Among the 247 female participants (median age, 53 years; mean body mass index [BMI], 23.7 kg/m^2^), 45.7% had disease progression or death during a median follow‐up of 36.4 months. After adjusting for age and visceral metastasis, SMD was the only independent predictor among body composition parameters for worse PFS (adjusted hazard ratio [HR] = 1.20 per standard deviation decrement, 95% CI: 1.01–1.42, *p* = 0.041), whereas BMI, muscle area, and fat area were not. Participants with low SMD had a higher risk of progression than those without (PFS, 27.2 vs. 51.1 months, *p* = 0.009; adjusted HR 1.84, 95% CI: 1.22–2.76, *p* = 0.003). Strong associations between low SMD and poor PFS were observed in groups with pre‐menopause status (HR, 3.04 vs. 1.19 in post‐menopause; 95% CI: 1.54–5.99, *p* for interaction < 0.05) and without visceral metastases (HR, 2.95 vs. 1.19 in with visceral metastases; 95% CI: 1.59–5.49, *p* for interaction < 0.05).

**Conclusions:**

CT‐defined low SMD predicts poor treatment outcomes in patients with ABC undergoing first‐line treatment with aromatase inhibitors and CDK4/6i.

## 1 Introduction

Hormone receptor‐positive and HER2‐negative breast cancers constitute 60%–70% of breast cancer cases [1] and show the highest dependence on the cyclin D‐CDK4/6 signalling pathway among breast cancer subtypes. CDK4/6 inhibitors (CDK4/6i) plus endocrine therapy (ET) have been established as a first‐line therapy for advanced hormone receptor‐positive and HER2‐negative breast cancer. Moreover, adjuvant abemaciclib or ribociclib treatment has shown evident invasive disease‐free survival gain in patients with surgically resected stage II or III breast cancer [2, 3], further expanding the CDK4/6i indication to include approximately 60% of all patients with hormone receptor‐positive and HER2‐negative breast cancer [4], representing approximately 700 000 new cases worldwide.

A recent study also identified several potential tumour genetic factors for CDK4/6i treatment response prediction, including *RB1*, *BRCA1*/*BRCA2*, and *CDKN2A/2B/2C* mutations and *FAT1* loss [5].^S1^ However, these mutations were found in only a small subset of patients with luminal breast cancer [5], and no definitive factor has been identified to govern therapeutic decisions regarding endocrine plus CDK4/6i treatment.

Aside from tumour genetic factors, other host factors influence the response to targeted therapy or immunotherapy, including cancer‐related cachexia, gut microbiota imbalance, and diurnal variations in glucocorticoid hormones [6, 7, 8]. These findings raise a potential connection between host metabolism and the mode of action of anti‐cancer therapy. In line with advancements in precision medicine, there is a growing need to closely examine host factors to improve treatment efficacy.

Patients with breast cancer with high body mass index (BMI) often have poor prognosis, probably related to their high level of tumour‐promoting systemic inflammation and insulin resistance [9]. However, a recent post‐hoc analysis of the phase III PALLAS adjuvant trial showed that patients with obesity had a low frequency of neutropenia on CDK4/6i treatment, which may be due to relatively low drug exposure [10]. Aside from a high BMI, components of body composition, such as muscle, abdominal visceral fat, subcutaneous fat, and bone, have a crucial prognostic impact in patients with cancer [11, 12]. Obesity‐related systemic inflammation is known to activate IGF and leptin signals that promote the growth of breast cancers and resistance to ET and CDK4/6i [13, 14]. Therefore, the implication of the body composition of patients in response to endocrine plus CDK4/6i therapy in patients with luminal breast cancer should be investigated. In this context, computed tomography (CT) body composition analysis enables convenient and reproducible body composition measurement of patients. We have previously generated and validated an artificial intelligence (AI)‐based semi‐automated body composition analysis model using lumbar 3 (L3) level CT cross‐sectional axial images [15].

In this study, we analysed patients with advanced hormone receptor‐positive and HER2‐negative breast cancer who received a CDK4/6i plus an aromatase inhibitor as the first‐line treatment at the Yonsei Cancer Center. We performed baseline L3 CT body composition analysis of these patients and evaluated the impact of difference in body composition indices and low skeletal muscle radiodensity (SMD) on survival outcomes after aromatase inhibitor plus CDK4/6i treatment.

## 2 Methods

### 2.1 Study Population and Data Collection

From a prospective metastatic breast cancer database (MBC‐DB, institutional review board [IRB] number: 4‐2016‐0574) at the Yonsei Cancer Center, we analysed patients with hormone receptor‐positive, HER2‐negative ABC who received CDK4/6i in combination with an aromatase inhibitor between January 2017 and October 2020. We excluded patients with an unverifiable baseline BMI, male sex, use of fulvestrant as a CDK4/6i partner, combination therapy with other investigational drugs, a history of treatment for other malignancies within the last 3 years, patients without non‐contrast images from baseline abdominopelvic (AP) CT or PET‐CT, and those without qualifying level images (CONSORT diagram, Figure S1).

This study was a retrospective secondary analysis that did not require informed consent beyond the initial acquisition of the MBC‐DB. This study was approved by the IRB of Yonsei University Health System (IRB number: 4‐2022‐1315).

### 2.2 Treatment

The patients were treated with CDK4/6i and aromatase inhibitors, following the recommended standard dosing regimen for each drug. This regimen included letrozole, exemestane, and anastrozole as aromatase inhibitors and palbociclib, ribociclib, and abemaciclib as CDK4/6i. Menopausal status was determined shortly before the initiation of first line aromatase inhibitor plus CDK4/6 inhibitor for advanced breast cancer, and all pre‐menopausal patients underwent either bilateral salpingo‐oophorectomy prior to the treatment or received a gonadotropin‐releasing hormone (GnRH) agonist for ovarian suppression. Dose adjustments or interruptions were made at the clinician's discretion based on the tolerability and adverse event profile of the treatment. The intervals between metastatic disease diagnosis, imaging, and treatment initiation were within 2 months in most cases (Figure S2). Tumour response was evaluated every 8–12 weeks following standard routine practice using radiographic scanning (e.g., chest/AP CT) based on clinical assessment. Treatment‐related adverse events were graded according to the Common Terminology Criteria for Adverse Events version 5.0.

### 2.3 Image Protocols and AI‐Based Body Composition Analysis

Baseline non‐contrast APCT or PET‐CT images of study participants were obtained. The dataset included images acquired up to 60 days before or 15 days after the start of CDK4/6i therapy. The CT protocols and specifications were as follows: manufacturers (GE Medical Systems, 75%; Siemens, 21%; Philips, 4%), kVp (120, 77%; 100, 18%; 110/130/140, 5%), and kernel (soft, 48%; standard, 27%; B30f, 13%; and other, 12%). A dedicated commercialized deep learning software (Deepcatch, v1.2.0.6619, MEDICALIP Co. Ltd., Seoul, Korea) was used to obtain body composition indices from the CT scans. This software allows the semiautomatic segmentation and quantification of different body composition components, such as abdominal visceral fat (AVF), subcutaneous fat (SF), skeletal muscle area (SMA), and radiation attenuation coefficient (SMD in Hounsfield Units [HU]). The software utilizes a deep neural network algorithm to segment and quantify bone, muscle, and fat in CT axial images, showing good accuracy (dice similarity coefficient for skeletal muscle and fat region: 0.97–0.99 compared with a standard reference generated by a human expert) and reproducibility (intraclass correlation using a two‐way random effects model: 0.99; 95% CI: 0.98–0.99) [15]. After the automated segmentation of L3 cross‐sectional images, manual inspection and correction for any potential errors in the segmentation process were performed to ensure segmentation quality. All images in this study were analysed by a single operator with more than 7 years of experience in image analysis.

### 2.4 Definitions

BMI (kg/m^2^) categories were defined and classified as follows: underweight (BMI < 18.5), normal weight (18.5 ≤ BMI ≤ 24.9), overweight (25 ≤ BMI ≤ 29.9), and obese (BMI ≥ 30). Body composition items corresponding to the area were standardized by dividing them by the square of the height. For the sex‐specific cutoffs of sarcopenia, the skeletal muscle index (SMI, SMA/[height in meters]^2^) < 39 cm^2^/m^2^ was used [16]. As there is no clear standard cutoff for other body composition variables validated in patients with cancer, we divided them into tertiles for categorical analysis. The lowest tertile of SMD, which corresponds to values ≤ 28.7 HU, was designated as indicative of low SMD. Visceral metastases were defined as the metastatic involvement of the liver and/or lungs. The neutrophil‐to‐lymphocyte ratio (NLR) was calculated using counts obtained from peripheral blood leukocytes, neutrophils, and lymphocytes within a timeframe of 15 days before or after day 28 following treatment initiation.

### 2.5 Statistical Analysis

Correlations between continuous variables were assessed using Pearson correlation coefficients. Progression‐free survival (PFS) was defined as the time from initiating CDK4/6i combined with ET to either radiologically confirmed disease progression or death. Overall survival (OS) was calculated from the commencement of treatment until patient death. Patient survival was monitored until March 2023, and the median follow‐up duration was derived using the reverse Kaplan–Meier method. The results were illustrated using Kaplan–Meier plots, and the log‐rank test was used to assess survival differences among the patient groups. Hazard ratios (HRs) were determined using the Cox proportional hazards regression model. All statistical analyses were performed using SPSS (version 26.0, IBM Corp., Armonk, New York, USA), R (version 4.2.2, The R Foundation, Vienna, Austria), and GraphPad Prism software (Version 8.0 for Windows, GraphPad Software, Boston, Massachusetts, USA).

## 3 Results

### 3.1 Patient Cohort and Baseline Characteristics

From January 2017 to October 2020, 456 patients who were diagnosed with hormone receptor‐positive, HER2‐negative ABC and treated with a CDK4/6i in combination with an aromatase inhibitor at the Yonsei Cancer Center were enrolled in this study (CONSORT diagram, Figure S1). The final analysis cohort included 247 patients, with a median follow‐up of 36.4 months (interquartile range [IQR], 19.5–49.5) as of 30 March 2023. At this cutoff, 123 patients underwent CDK4/6i plus ET. The median age of the patient cohort was 53 years (IQR: 47.0–61.5 years). Most patients were Asian (98.0%) and endocrine‐sensitive or endocrine‐naïve (74.1%). In the cohort, 104 pre‐menopausal patients had either undergone surgical menopause or received ovarian suppression with a GnRH agonist. The bilateral salpingo‐oophorectomy was performed in 60 premenopausal patients for ovarian function suppression. The median time interval from bilateral salpingo‐oophorectomy to CDK4/6 inhibitor initiation was 10.5 days, and 90% (54/60) of patients received the surgery within 35 days before treatment initiation. In addition, 135 (54.7%) patients exhibited visceral metastases. Detailed information regarding the disease characteristics and baseline body composition parameters is presented in Table 1.

**TABLE 1:** Baseline characteristics of the study participants.

	Patients (*N* = 247)	Low‐SMD^b^	Normal‐SMD		Pre‐menopause	Post‐menopause	
		(*n* = 83)	(*n* = 164)	*p*	(*n* = 103)	(*n* = 144)	*p*
Age, median [IQR], years	53 [47.0–61.5]	60 [52.0–72.5]	51 [45.0–58.0]	< 0.001	46 [41.0–49.5]	60 [54.0–70.3]	< 0.001
Age group, No. (%)							
< 50 years	92 (37.2)	16 (19.3)	76 (46.3)	< 0.001	77 (74.8)	15 (10.4)	< 0.001
≥ 50 years	155 (62.8)	67 (80.7)	88 (53.7)		26 (25.2)	129 (89.6)	
BMI, mean ± SD, kg/m^2^	23.7 ± 3.4	24.5 ± 3.6	23.2 ± 3.3	0.008	23.2 ± 3.0	24.0 ± 3.7	0.085
Ethnicity, No. (%)							
Asian	242 (98.0)	82 (98.8)	160 (97.6)	0.086	103 (100.0)	139 (96.5)	0.146
White	5 (2.0)	1 (1.2)	4 (2.4)		—	5 (3.5)	
De novo stage IV, No. (%)	99 (40.1)	35 (42.2)	64 (39.0)	0.735	40 (38.8)	59 (41.0)	0.837
Visceral metastasis, No. (%)	135 (54.7)	41 (49.4)	94 (57.3)	0.296	57 (55.3)	78 (54.2)	0.958
Endocrine resistance, No. (%)							
Naïve	183 (74.1)	68 (81.9)	115 (70.1)	0.035	58 (56.3)	125 (86.8)	< 0.001
Primary	18 (7.3)	7 (8.5)	11 (6.7)		9 (8.7)	9 (6.2)	
Secondary	46 (18.6)	8 (9.6)	38 (23.2)		36 (35.0)	10 (7.0)	
Menopause, No. (%)							
Pre	103 (41.7)	19 (22.9)	84 (51.2)	< 0.001	—	—	
Post	144 (58.3)	64 (77.1)	80 (48.8)		—	—	
BSO, No. (%)	60 (24.3)	13 (15.6)	47 (28.6)	0.036	56 (54.4)	4 (2.8)	< 0.001
Type 2 DM, No. (%)	32 (13.0)	18 (21.7)	14 (8.5)	0.007	7 (6.8)	25 (17.4)	0.025
L3–SMI, median [IQR], cm^2^/m^2^	40.2 [36.4–44.3]	40.0 [36.0–45.0]	40.3 [36.9–43.9]	0.991	41.7 [37.1–45.8]	39.9 [36–43.2]	0.042
L3–AVFI, median [IQR], cm^2^/m^2^	33.2 [18.4–46.2]	39.8 [30.0–55.0]	28.2 [15.3–41.4]	< 0.001	26.2 [16.9–37.1]	37.8 [22.9–51.9]	< 0.001
L3–SFI, median [IQR], cm^2^/m^2^	68.5 [52.4–107.1]	70.0 [57.0–91.4]	68.3 [50.6–87.1]	0.164	68.3 [51.9–90.2]	69.5 [53.6–86.7]	0.750
L3–SMD, median [IQR], HU	33 [26–39]	23 [17–26]	37 [33–41]	< 0.001	37 [32–41]	30 [23–35]	< 0.001
Sarcopenia,^a^ No. (%)	104 (42.1)	35 (42.2)	69 (42.1)	1.000	39 (37.9)	65 (45.1)	0.312
Low–SMD, No. (%)	83 (33.6)	—	—		19 (18.4)	64 (44.4)	< 0.001

Abbreviations: AVFI, abdominal visceral fat index; BMI, body mass index; BSO, bilateral salpingo‐oophorectomy; DM, diabetes mellitus; IQR, interquartile range; L3, third lumbar spine vertebra; SD, standard deviation; SFI, subcutaneous fat index; SMD, skeletal muscle radiodensity; SMI, skeletal muscle index.

^a^ Defined as SMI < 39 cm^2^/m^2^.

^b^ Defined as low tertile group.

### 3.2 Baseline Body Composition Analysis of the Patient Cohort

**FIGURE 1:**
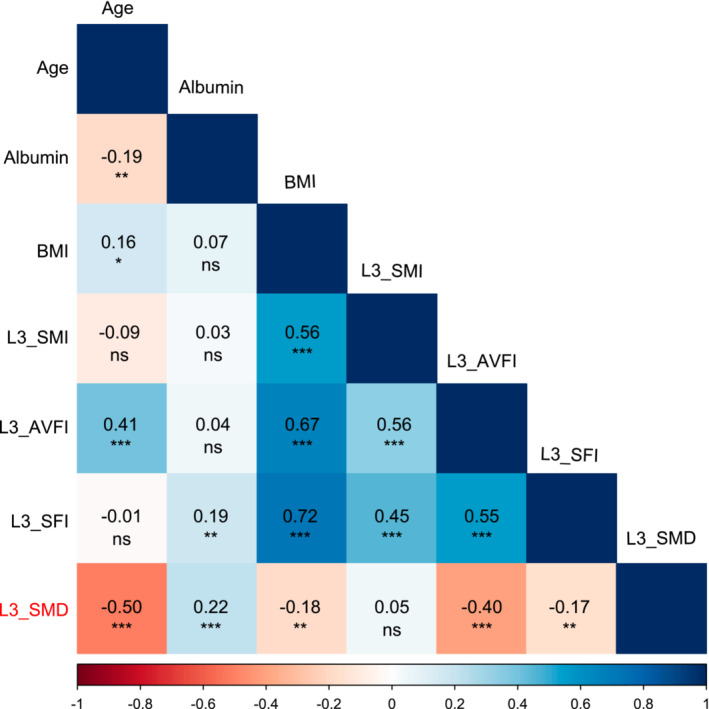
Correlation matrix of body composition parameters and clinical factors. Each cell in the matrix represents the correlation coefficient with the colour intensity and direction (blue for positive, red for negative) indicating the strength and direction of the correlation. Statistical significance of correlations is noted with asterisks (*p < 0.05; **p < 0.01; ***p < 0.001, ns, not significant).

Using baseline L3 level non‐enhanced CT scan images before the initiation of aromatase inhibitor plus CDK4/6i treatment, the body composition parameters (SMI, subcutaneous fat index [SFI], abdominal visceral fat index [AVFI], and SMD) were estimated in all patients. The median BMI was 23.4 kg/m^2^ (IQR, 21.5–25.0), and 23.4% of patients were overweight or obese. The median SMI was 40.2 kg/m^2^ (IQR, 36.4–44.3), with 42.1% having sarcopenia. The BMI of our patient cohort was lower than that in Western studies, but the SMI was comparable [17]. The analysis of the relationship between body composition parameters and clinical characteristics revealed that SMI, AVFI, and SFI were significantly and positively correlated with BMI (Figure 1). Younger patients had a higher SMD than older patients, consistent with previous studies [18, 19]. The SMD showed a weak positive correlation with serum albumin levels and a moderate negative correlation with AVFI, along with a weak negative correlation with BMI. There was no significant correlation between the SMD and SMI.

### 3.3 Association Between Body Composition Metrics and PFS

To further explore which aspects of body composition significantly influenced the long‐term response to CDK4/6i, we conducted a PFS analysis using body composition parameters. Only SMD was significantly associated with PFS. An SMD value lower by one standard deviation (SD, 11 HU) correlated with worse PFS (HR, 1.18; 95% CI: 1.01–1.38; *p* = 0.038) (Table 2). The multivariate analysis adjusted for patient age and visceral metastases revealed lower SMD as an independent negative predictive factor for PFS on CDK4/6i plus aromatase inhibitor treatment (HR, 1.20; 95% CI: 1.01–1.42; *p* = 0.041) (Table 2).

**TABLE 2:** Factors associated with progression‐free survival.

Variable		Univariable		Multivariable	
		HR (95% CI)	*p*	HR (95% CI)	*p*
Age, by 5 years		1.01 (0.93–1.08)	0.89	0.97 (0.89–1.05)	0.39
Baseline BMI	< 25	Reference			
	≥ 25	1.01 (0.66–1.55)	0.95		
De novo stage IV	Yes	0.77 (0.52–1.13)	0.18		
Visceral metastasis	Yes	1.59 (1.08–2.32)	0.017^a^	1.53 (1.04–2.25)	0.030^a^
Endocrine resistance	Primary	1.80 (0.96–3.39)	0.07		
	Secondary	1.35 (0.83–2.18)	0.22		
Menopause	Pre	Reference			
	Post	1.34 (0.91–1.99)	0.14		
Neutropenia ≥ G3	Yes	0.86 (0.59–1.25)	0.43		
L3–SMI, per 1SD decrement		0.93 (0.77–1.12)	0.43		
L3–AVFI, per 1SD decrement		1.05 (0.88–1.25)	0.60		
L3–SFI, per 1SD decrement		1.03 (0.85–1.25)	0.76		
L3–SMD, per 1SD decrement		1.18 (1.01–1.38)	0.038^a^	1.20 (1.01–1.42)	0.041^a^

Abbreviations: AVFI, abdominal visceral fat index; BMI, body mass index; DM, diabetes mellitus; L3, third lumbar spine vertebra; SD, standard deviation; SFI, subcutaneous fat index; SMD, skeletal muscle radiodensity; SMI, skeletal muscle index.

^a^ *p*‐value < 0.05.

**FIGURE 2:**
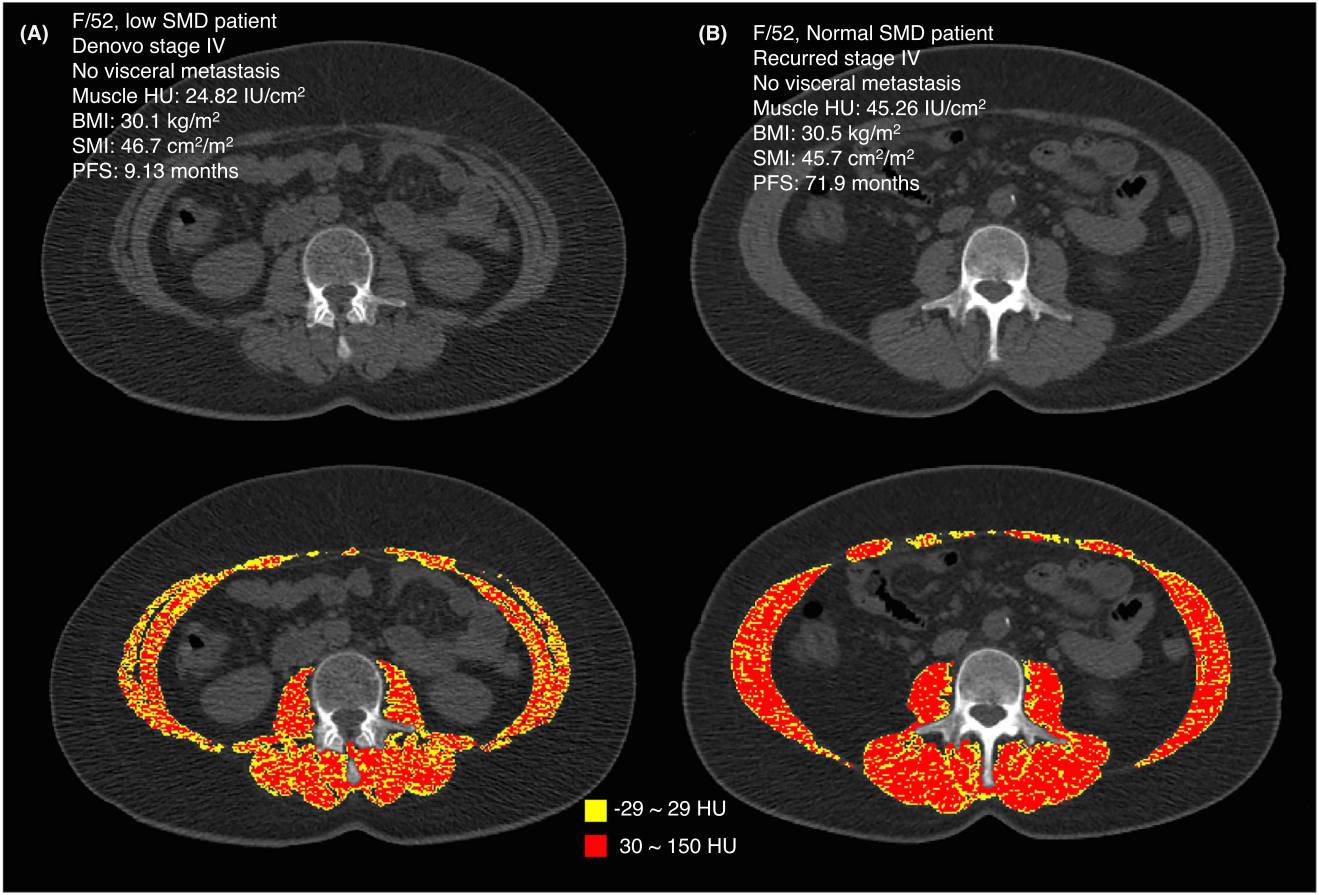
Non‐enhanced axial abdominopelvic computed tomography (APCT) image with a Hounsfield unit (HU)‐based colour scale of the skeletal muscles of (A) a patient with low skeletal muscle radiodensity (SMD) and (B) a patient with normal SMD. Both patients are of the same age, have similar skeletal muscle index (SMI) and body mass index (BMI) profiles, and are without visceral metastasis. Lower muscle attenuation, represented by a HU range of −29 to 29, is depicted in yellow, while normal muscle attenuation, defined by a HU range of 30 to 150, is illustrated in red.

**FIGURE 3:**
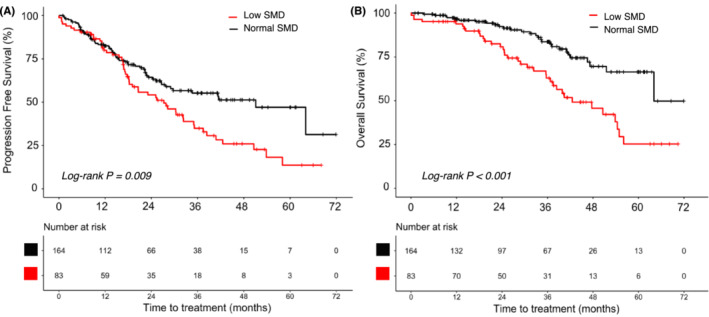
Analysis comparing (A) progression‐free survival and (B) overall survival by skeletal muscle radiodensity (SMD) status.

Patients with low SMD were older and had a higher proportion of post‐menopausal women (Table 1). This is because muscle radiodensity generally correlated negatively with patient age; however, 18.4% of pre‐menopausal patients were also classified as having low SMD (Figure S3). Relatively increased fat infiltration in the skeletal muscles was observed in patients with low SMD, even when their BMI and SMA were similar (Figure 2). Multivariate Cox regression analysis adjusted for age and visceral metastases showed that patients with low SMD exhibited an 84% higher risk of disease progression (HR, 1.84; 95% CI: 1.22–2.76; *p* = 0.003) (Table S1). Patients with low SMD also showed significantly shorter PFS (27.2 vs. 51.1 months; HR, 1.63; 95% CI: 1.13–2.36; *p* = 0.009) and OS (42.4 vs. 64.1 months; HR 2.58; 95% CI: 1.57–4.22; *p* < 0.001) than patients with higher tertile muscle radiodensities (Figure 3).

### 3.4 Implications of Low SMD in Pre‐Menopausal Individuals and Patients With Non‐Visceral Metastases

**FIGURE 4:**
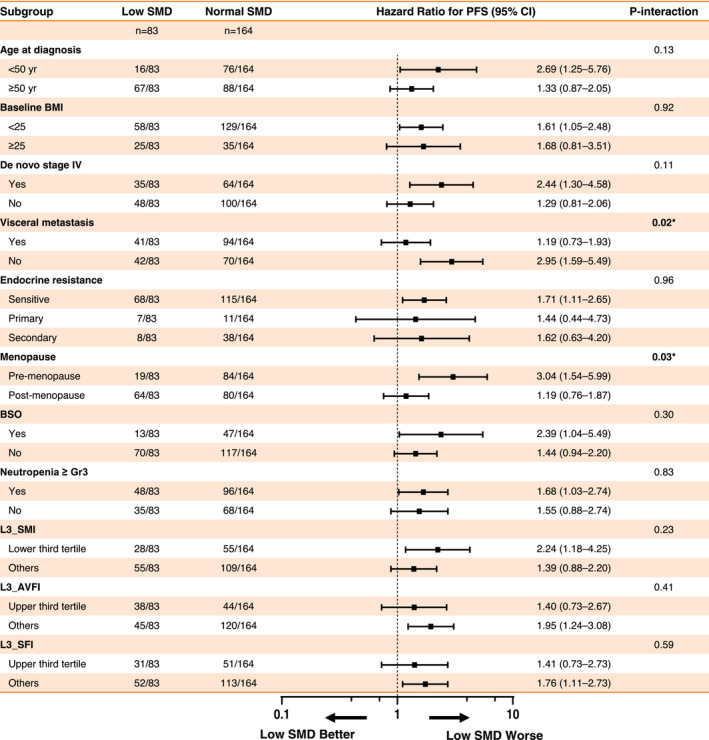
Forest plot for PFS illustrating a low skeletal muscle radiodensity (SMD) subgroup analysis of various clinical factors. The number of patients for each corresponding subgroup is described.

**FIGURE 5:**
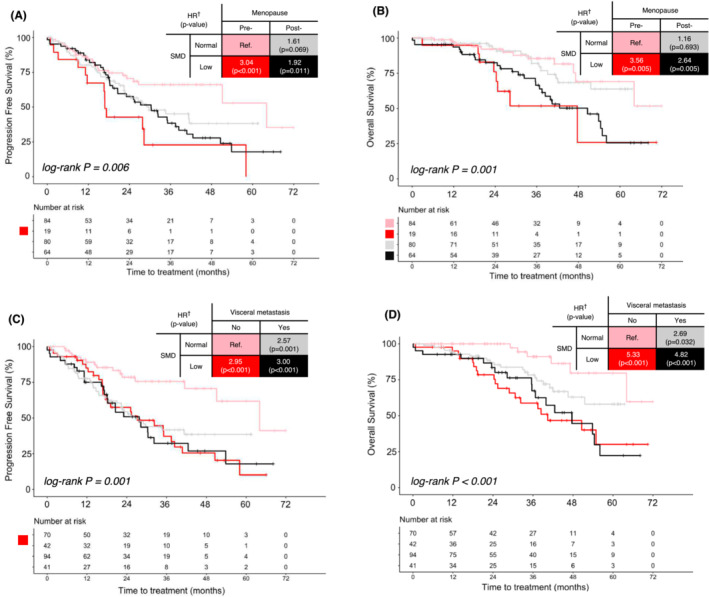
Low skeletal muscle radiodensity (SMD) subgroup analyses of special interest. Stratified by menopausal status (A, progression‐free survival [PFS]; B, overall survival [OS]) and visceral metastasis (C, PFS; D, OS).

Next, we performed a PFS analysis for low SMD across patient subgroups. The impact of low SMD on PFS showed a statistically significant interaction with menopausal status and visceral metastasis, suggesting that these factors were crucial for interpreting low SMD (Figure 4). The swimmer plot revealed that patients with low SMD exhibited a notably higher rate of disease progression in both the pre‐menopausal cohort and those without visceral metastases. BMI was not associated with muscle radiodensity status (Figures S4,S5). Low SMD was associated with poor PFS only in pre‐menopausal individuals (HR, 3.04; 95% CI: 1.54–5.99), not in post‐menopausal patients (HR, 1.19; 95% CI: 0.76–1.87) (Figure 5A,B, Table S2). Similarly, patients with low SMD showed worse PFS (27.2 vs. 64.1 months; HR, 2.94; 95% CI: 1.58–5.45; *p* < 0.001) than normal SMD patients in the non‐visceral metastases subgroup (Figure 5C,D, Table S2).

### 3.5 Metabolic and Inflammatory Markers in Low SMD

We also investigated the association of low SMD with metabolic and inflammatory markers. Individuals with low SMD exhibited a significantly higher on‐treatment NLR at the beginning of the second cycle (28 ± 15 d from treatment initiation) (*p* < 0.001; Figure S6A). However, we observed no significant differences in baseline serum triglyceride (TG), C‐reactive protein (CRP), and triglyceride and glucose (TyG) indices (Figure S6B–D).

### 3.6 Association Between Body Composition Metrics and Neutropenia

The PALLAS trial demonstrated a lower incidence of neutropenia with CDK4/6i and aromatase inhibitor as adjuvant treatment in patients with BMI ≥ 25 without affecting treatment efficacy [10]. In our study, 144 patients (58.3%) developed grade 3 or higher neutropenia within the first 28 days of starting CDK4/6i in combination with aromatase inhibitors. Our patient population showed a relatively low incidence of BMI ≥ 25 (24.3%), and there was no statistical correlation between BMI ≥ 25 and the incidence of grade 3 or higher neutropenia (*p* = 0.89). However, multivariate logistic regression analysis showed that being in the upper third tertile of AVFI significantly reduced the risk of developing neutropenia (adjusted odds ratio, 0.49; 95% CI: 0.26–0.94; *p* = 0.03; Table S3).

## 4 Discussion

Our study demonstrated that CT‐estimated low SMD at baseline robustly predicts unfavourable survival outcomes with aromatase inhibitors plus CDK4/6i in patients with luminal breast cancer. In contrast to the BMI, low SMD is an indicator of decreased physical function and increased systemic inflammation in cancer patients. This suggests a potential connection between host body composition status and the sensitivity of breast cancer cells to ET and CDK4/6i. Our subgroup analysis further revealed that low SMD was selectively associated with poor PFS in the pre‐menopausal and non‐visceral metastases subgroups, suggesting that decreased muscle density in young patients was particularly detrimental to CDK4/6i responses. Our study is the first to suggest that host body composition status plays a significant role in determining the response to CDK4/6 therapy, underscoring the necessity of integrating host body composition assessment into the future clinical practice. Additionally, we suggest that low SMD may be especially relevant in Asian populations, which typically have a low rate of obesity, as defined by conventional BMI cutoffs.

Low SMD reflects impaired physical function and condition named as ‘myosteatosis’ characterized by excessive adipose tissue infiltration into skeletal muscles [20],^S2^ resulting in decreased muscle radiodensity on CT scans [21]. Although direct measurement of tissue triglyceride content has not been performed, a previous study reported a correlation between CT‐derived skeletal muscle attenuation and muscle lipid content, as confirmed by percutaneous biopsy, leading most studies to define CT‐derived low SMD as indicative of myosteatosis [22, 23].^S3^ We suggest CT‐derived low SMD would be useful surrogate for myosteatosis, although it requires careful interpretation. Low SMD reflects obesity and insulin resistance, which are often associated with physical inactivity, increasing the risk of metabolic syndrome [23]. Recent studies have also reported potential prognostic implications of low SMD in patients with chronic inflammatory diseases and cancer [18, 24, 25]. Martin et al. reported that low muscle attenuation was associated with poor survival in 1473 patients with cancer regardless of their BMI [18]. In our study, we assessed CT‐obtained body composition markers, such as SMI, AVFI, SFI, and SMD, for outcomes related to ET and CDK4/6i treatments. Remarkably, SMD was the robust body composition predictor of PFS in addition to visceral metastasis in patients treated with CDK4/6i, highlighting its biological relevance.

Previous studies have evaluated the influence of sarcopenia and low SMD on breast cancer survival outcomes. Caan et al. found sarcopenia and high adiposity to be significant poor prognostic markers in non‐metastatic breast cancer, but muscle density was irrelevant for survival outcomes [11]. However, their study included a diverse group of patients in terms of cancer subtype and stage and did not focus on responses to specific systemic breast cancer therapy agents. Notably, in our analysis, low SMD was decoupled from low SMI, which is related to sarcopenia. Low SMD is distinct from sarcopenia because low SMD patients have lower muscle strengths and higher frailty scores than those with sarcopenia, even when their muscle masses are similar (Figure 2) [26, 27]. We suggest that low SMD may outperform conventional sarcopenia markers for therapy response prediction because it reflects the extent of adipose tissue infiltration to muscles and increased adipose‐related inflammation which may be related to the pharmacokinetic and pharmacodynamic properties of drugs. In line with our findings, Rier et al. also demonstrated that low muscle attenuation serves as a prognostic factor in metastatic breast cancer receiving first‐line palliative chemotherapy.

Low SMD and myosteatosis was originally reported as a prognostic factor for chronic diseases [28]. It leads to physical dysfunction by converting type II muscle fibres to type I fibres, which increases the risk of falls, frailty, and the development of cardiovascular disease [29]. Although a complete understanding of factors defining low SMD remains elusive, emerging evidence has linked it to insulin resistance and abnormal insulin signalling, including the activation of leptin‐IGF‐1 signalling that is also reported to cause CDK4/6i resistance preclinically [13, 30].^S3^ These factors contribute to increased systemic inflammation and oxidative stress. This cascade is believed to culminate in reduced protein synthesis and impaired functionality in target organs [31, 32]. In our cohort of patients with low SMD, we observed a statistically significant increase of on‐treatment NLR (*p* < 0.001), indicating heightened systemic inflammation [33, 34]. These findings are consistent with our previous work, which established NLR as a negative predictor of PFS in patients receiving palbociclib and endocrine treatment based on data from a multicenter retrospective cohort and the PALOMA‐2/3 studies [35]. We propose that patients with low SMD may be at higher risk of disease progression due to insulin resistance pathways and systemic inflammation that promote breast cancer cell growth and resistance to CKD4/6i treatment [13, 30].^S4^

In this study, we observed that low SMD had a profound predictive effect in pre‐menopausal and non‐visceral metastasis patients. Pre‐menopausal patients were metabolically distinct from post‐menopausal patients, and there was a negative correlation between age and SMD in our cohort. Therefore, low SMD in pre‐menopausal patients may indicate severe metabolic dysregulation, resulting in a clear demarcation of PFS between patients with and without low SMD. Based on preclinical studies, we propose that patients with low SMD have low systemic oestrogen levels [36].^S5^ This could imply that tumours in patients with low SMD are less reliant on oestrogen and the cyclin D‐CDK4/6 pathway. We also should note that all premenopausal patients in this study underwent ovarian suppression, which could induce abrupt metabolic changes, enhancing prognostic influence of low SMD. Traditionally, patients without visceral metastases have a more favourable prognosis than those with visceral metastases; however, these patients demonstrate a greater risk of low SMD. The identification of low SMD in patients without visceral metastases may indicate a risk level equivalent to that associated with visceral metastases. It is conceivable that the survival of patients with visceral metastases is influenced more by tumour genetic factors, whereas those with non‐visceral metastases might be more susceptible to host body composition factors. However, these hypothetical explanations should be thoroughly evaluated in future studies. Currently, we note that a potentially modifiable host factor, low SMD, may hold great significance in pre‐menopausal and non‐visceral metastatic patients.

Whether improving muscle composition status in patients with luminal breast cancer will result in improved prognostic outcomes is yet to be determined. Unlike visceral metastasis, low SMD is a modifiable host factor. In patients with overt Cushing syndrome, adrenalectomy to correct cortisol excess resulted in a trend toward improvement in both SMD and SMA [37]. This suggests that SMD may be modifiable and a more sensitive marker of muscle recovery. Recent studies suggest that increased physical activity combined with dietary interventions, such as vitamin D, branched‐chain amino acids, and beta‐hydroxy‐beta‐methylbutyrate, may improve muscle strength [38, 39]. To date, no pharmacological intervention trials have specifically targeted low SMD. However, exploring the effectiveness of combining therapies that potentially enhance insulin sensitivity, such as GLP‐1 agonists, GLT2 inhibitors, and exercise measures, with standard cancer treatments to improve response in specific patient groups is a promising area for future research.

This study analysed body composition parameters and their prognostic correlations in a distinct group of East Asian patients with hormone‐positive breast cancer receiving CDK4/6 and aromatase inhibitors, including pre‐menopausal individuals, a major strength of this study. Additionally, in Asian populations with a relatively low proportion of individuals with a BMI > 25, the AVFI appears to be a more reliable indicator of neutropenia than BMI in patients treated with CDK4/6 and aromatase inhibitors. However, this study has the typical limitations of a retrospective study design and, for some patients, a limited follow‐up period. Another important caveat is the partial volume effect in CT images, where tissues with different attenuation properties are captured in the same voxel, resulting in averaged values that may not accurately reflect tissue composition. Additionally, there may be variations in radiodensity depending on the measurement level [40], despite using a consistent method based on a single protocol to minimize such discrepancies. Nevertheless, we believe that these partial volume effect and variation would be normalized by using a consistent measurement method and applying it to a large population, thus preserving the significance of the association between low SMD and the response to CDK4/6 inhibitors. Lastly, we should note that since there is no consensus on definitive cutoff values for body composition parameters like SMD, we used tertiles in our analysis to minimize bias from arbitrarily selected cutoff points. We observed a minimal difference between the SMD cutoff of 28.7 (HU), derived from the tertile division, and the SMD cutoff of 33 (HU), calculated using the maximally selected log‐rank statistics.

In conclusion, our study provides novel insights into the robust impact of body composition differences, specifically low SMD, on the survival outcomes of first‐line aromatase inhibitor plus CDK4/6i treatment in patients with ABC. Patients with low SMD had an 83.8% increased risk of progression or death compared with those without low SMD; this impact was more pronounced in the pre‐menopausal and non‐visceral metastasis individuals. Our study highlights the role of host body composition in the therapy response to endocrine plus CDK4/6i therapy, which should be incorporated into future clinical practice and trial designs. Our findings also advocate for further investigation into the strategic management of low SMD through tailored nutritional, pharmacological, and exercise measures, offering potential avenues for improving treatment outcomes in patients with luminal breast cancer.

## Conflicts of Interest

The authors declare no conflicts of interest.

## Supporting information


**Data S1.** Supporting Information.

**Figure S1.** Consort diagram.
**Figure S2.** Timeline from metastatic disease diagnosis and CT/PET‐CT imaging to treatment initiation.
**Figure S3.** Relationship between age and L3‐skeletal muscle radiodensity (SMD) in the context of menopausal status and its impact on disease progression.
**Figure S4.** Swimmer plot of the outcomes in the without visceral metastasis subgroup, divided into two groups based on a BMI cut‐off of 25 kg/m^2^ and categorized by skeletal muscle radiodensity (SMD) status.
**Figure S5.** Swimmer plot of the outcomes in the premenopausal subgroup, divided into two groups based on a BMI cut‐off of 25 kg/m^2^ and categorized by skeletal muscle radiodensity (SMD) status.
**Figure S6.** Comparative analysis of metabolic and inflammatory markers based on skeletal muscle radiodensity (SMD). (A) Neutrophil‐to‐lymphocyte ratio, (B) triglyceride/glucose index, (C) triglyceride, and (D) C‐reactive protein.

**Table S1.** Univariate and multivariate analyses of factors associated with progression‐free survival categorized by body composition parameters (lower tertile group).
**Table S2.** Hazard ratio (HR) and p value summary for progression‐free survival (PFS) and overall survival (OS) between normal skeletal muscle radiodensity (SMD) and low SMD in premenopausal, postmenopausal, with VM, and without VM subgroups.
**Table S3.** Body composition parameters adjusted for age and body mass index (BMI) in relation to grade 3 or higher neutropenia.
**Table S4.** Number and percentage of patients classified as having sarcopenia based on different skeletal muscle index (SMI) cut‐off values.

## Data Availability

Requests for data sharing related to this study require prior consent of the corresponding author and must follow the established procedures and policies of Yonsei Cancer Center.
