# Supplementary material for: Low Skeletal Muscle Radiodensity Predicts Response to CDK4/6 Inhibitors Plus Aromatase Inhibitors in Advanced Breast Cancer
Source: J Cachexia Sarcopenia Muscle. 2024 Dec 17;16(1):e13666. doi: 10.1002/jcsm.13666 (PMC11670169; doi:10.1002/jcsm.13666)
Supplement: Supplementary file 1 — Data S1. Supporting Information. [file JCSM-16-e13666-s002.docx]

**Supplementary reference**

S1. Li Z, Razavi P, Li Q, Toy W, Liu B, Ping C, et al. Loss of the FAT1 Tumor Suppressor Promotes Resistance to CDK4/6 Inhibitors via the Hippo Pathway. Cancer Cell 2018;34:893-905.e898.

S2. McGovern J, Golder AM, Dolan RD, Roxburgh CSD, Horgan PG, McMillan DC. The combination of computed tomography‐derived muscle mass and muscle density and relationship with clinicopathological characteristics and survival in patients undergoing potentially curative surgery for colorectal cancer. JCSM Clinical Reports 2022;7:65-76.

S3. Kim MJ, Cho YK, Jung HN, Kim EH, Lee MJ, Jung CH, et al. Association Between Insulin Resistance and Myosteatosis Measured by Abdominal Computed Tomography. J Clin Endocrinol Metab 2023;108:3100-3110.

S4. Yee LD, Mortimer JE, Natarajan R, Dietze EC, Seewaldt VL. Metabolic Health, Insulin, and Breast Cancer: Why Oncologists Should Care About Insulin. Frontiers in Endocrinology 2020;11.

S5. Leite RD, Prestes J, Bernardes CF, Shiguemoto GE, Pereira GB, Duarte JO, et al. Effects of ovariectomy and resistance training on lipid content in skeletal muscle, liver, and heart; fat depots; and lipid profile. Appl Physiol Nutr Metab 2009;34:1079-1086.
