# Supplementary material for: Low Skeletal Muscle Radiodensity Predicts Response to CDK4/6 Inhibitors Plus Aromatase Inhibitors in Advanced Breast Cancer
Source: J Cachexia Sarcopenia Muscle. 2024 Dec 17;16(1):e13666. doi: 10.1002/jcsm.13666 (PMC11670169; doi:10.1002/jcsm.13666)
Supplement: Supplementary file 2 — Figure S1. Consort diagram. Figure S2. Timeline from metastatic disease diagnosis and CT/PET‐CT imaging to treatment initiation. Figure S3. Relationship between age and L3‐skeletal muscle radiodensity (SMD) in the context of menopausal status and its impact on disease progression. Figure S4. Swimmer plot of the outcomes in the without visceral metastasis subgroup, divided into two groups based on a BMI cut‐off of 25 kg/m2 and categorized by skeletal muscle radiodensity (SMD) status. Figure S5. Swimmer plot of the outcomes in the premenopausal subgroup, divided into two groups based on a BMI cut‐off of 25 kg/m2 and categorized by skeletal muscle radiodensity (SMD) status. Figure S6. Comparative analysis of metabolic and inflammatory markers based on skeletal muscle radiodensity (SMD). (A) Neutrophil‐to‐lymphocyte ratio, (B) triglyceride/glucose index, (C) triglyceride, and (D) C‐reactive protein. [file JCSM-16-e13666-s001.pptx]

## Slide 1
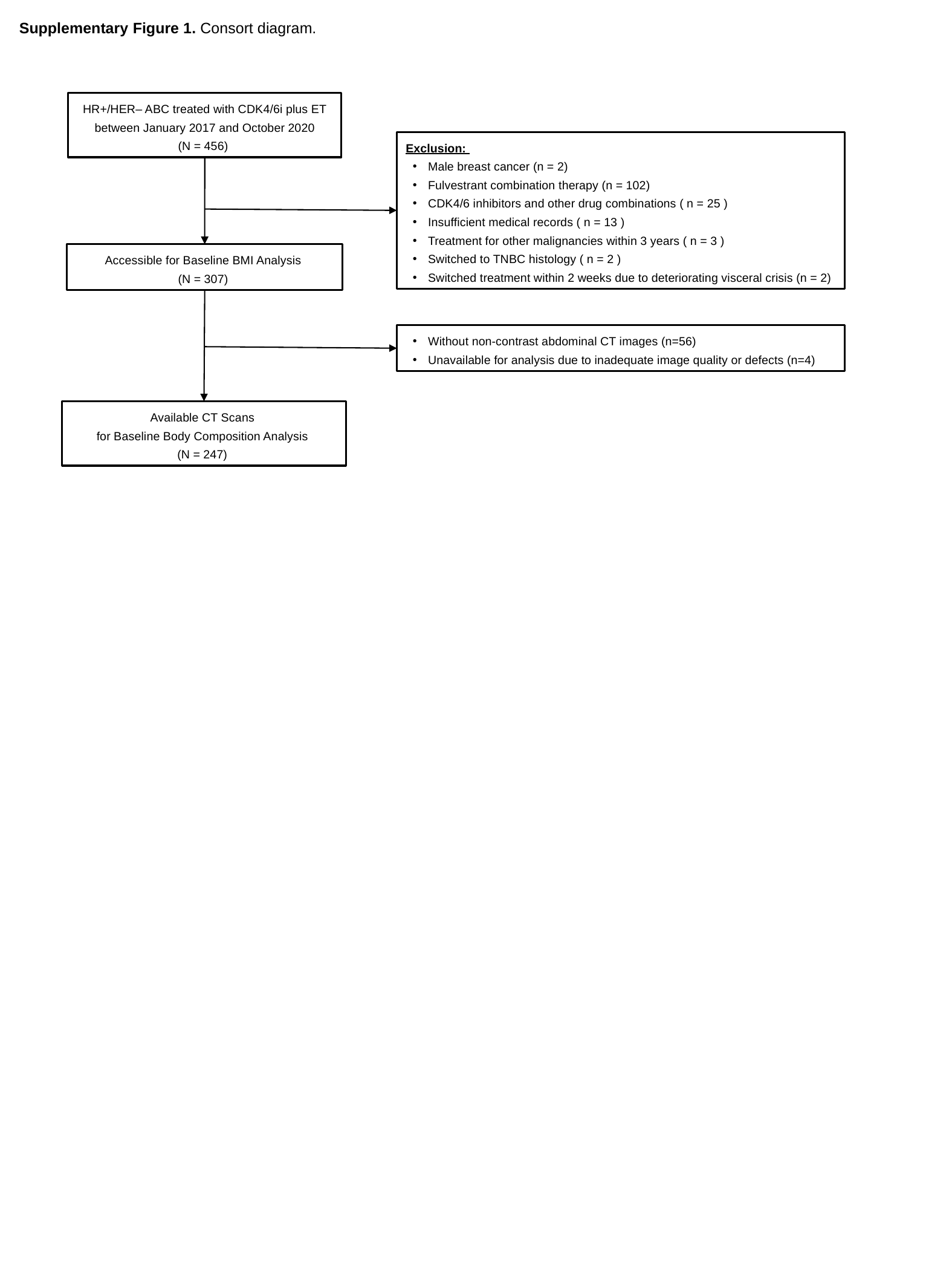

Supplementary Figure 1. Consort diagram.
HR+/HER– ABC treated with CDK4/6i plus ET
between January 2017 and October 2020
(N = 456)
Exclusion:
Male breast cancer (n = 2)
Fulvestrant combination therapy (n = 102)
CDK4/6 inhibitors and other drug combinations ( n = 25 )
Insufficient medical records ( n = 13 )
Treatment for other malignancies within 3 years ( n = 3 )
Switched to TNBC histology ( n = 2 )
Switched treatment within 2 weeks due to deteriorating visceral crisis (n = 2)
Accessible for Baseline BMI Analysis
(N = 307)
Without non-contrast abdominal CT images (n=56)
Unavailable for analysis due to inadequate image quality or defects (n=4)
Available CT Scans
for Baseline Body Composition Analysis
(N = 247)

## Slide 2
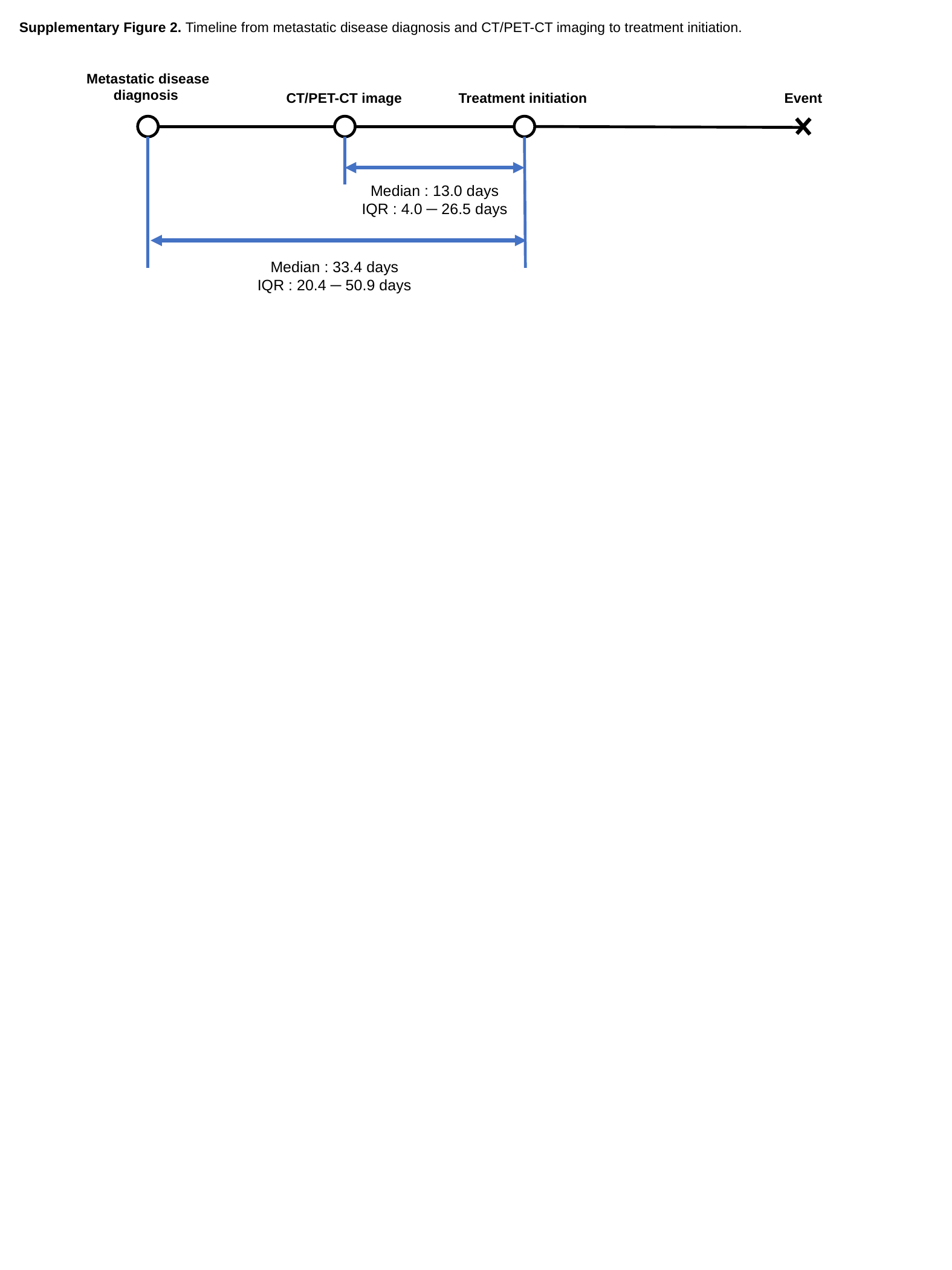

Supplementary Figure 2. Timeline from metastatic disease diagnosis and CT/PET-CT imaging to treatment initiation.
Metastatic disease diagnosis
CT/PET-CT image
Treatment initiation
Event
Median : 13.0 days
IQR : 4.0 ─ 26.5 days
Median : 33.4 days
IQR : 20.4 ─ 50.9 days

## Slide 3
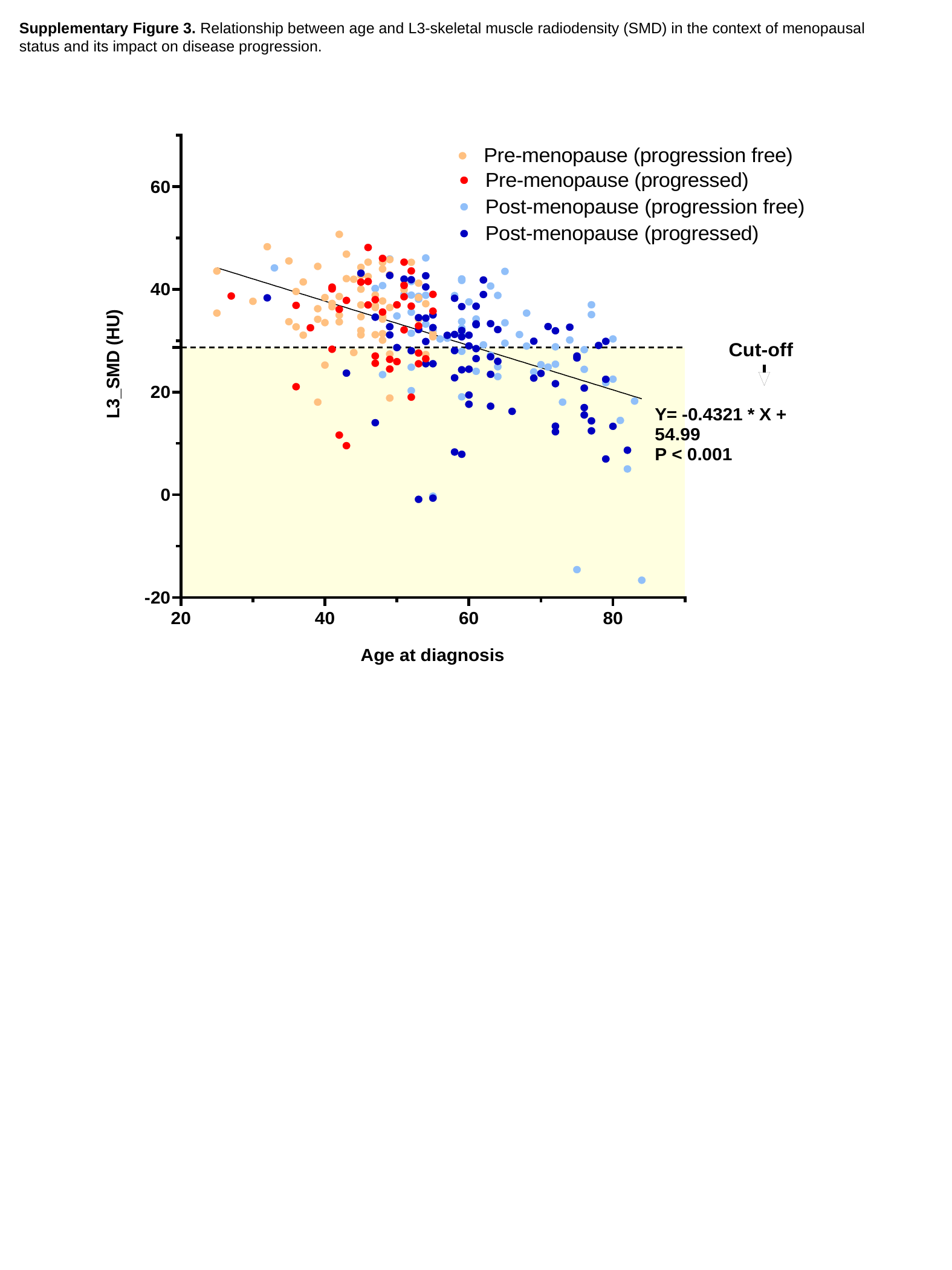

Supplementary Figure 3. Relationship between age and L3-skeletal muscle radiodensity (SMD) in the context of menopausal status and its impact on disease progression.

## Slide 4
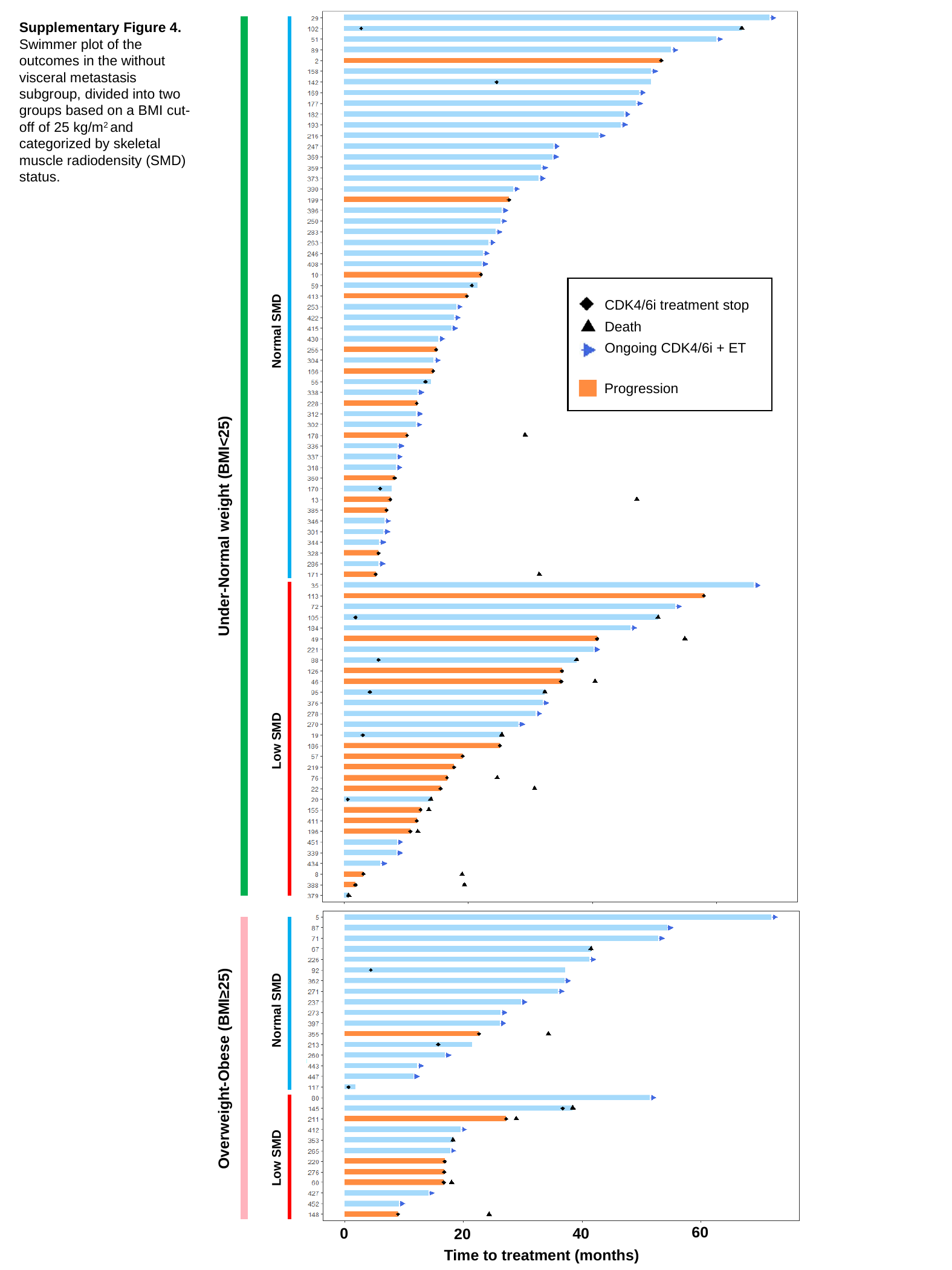

Supplementary Figure 4. Swimmer plot of the outcomes in the without visceral metastasis subgroup, divided into two groups based on a BMI cut-off of 25 kg/m2 and categorized by skeletal muscle radiodensity (SMD) status.
CDK4/6i treatment stop
Death
Ongoing CDK4/6i + ET
Progression
Normal SMD
Under-Normal weight (BMI<25)
Low SMD
Overweight-Obese (BMI≥25)
Normal SMD
Low SMD
60
0
40
20
Time to treatment (months)

## Slide 5
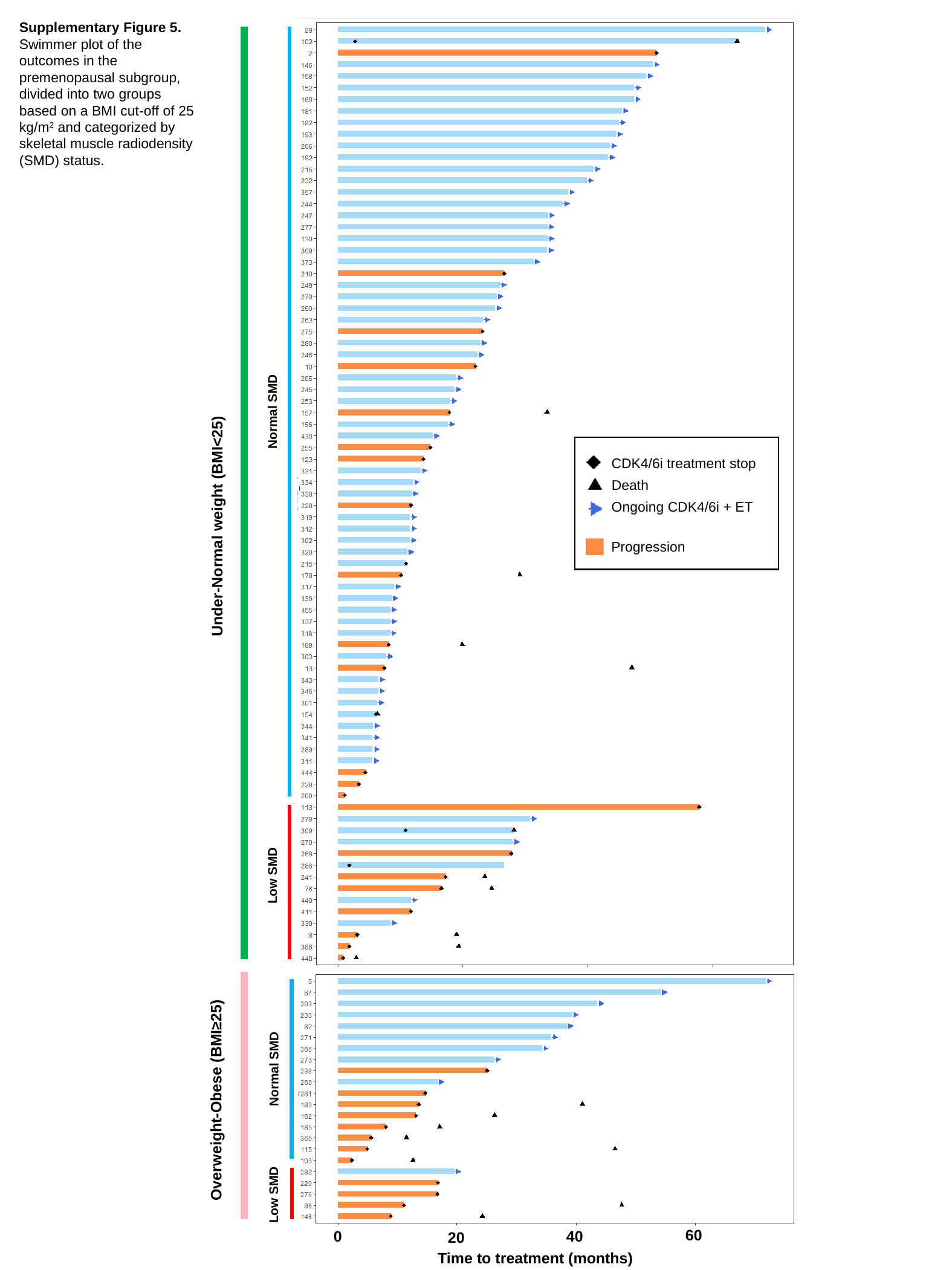

BMI group 을 왼쪽 bar 로 빼고
Swimmer plot bar color 를 progression 여부로 변경
Supplementary Figure 5. Swimmer plot of the outcomes in the premenopausal subgroup, divided into two groups based on a BMI cut-off of 25 kg/m2 and categorized by skeletal muscle radiodensity (SMD) status.
Normal SMD
Under-Normal weight (BMI<25)
CDK4/6i treatment stop
Death
Ongoing CDK4/6i + ET
Progression
Low SMD
Overweight-Obese (BMI≥25)
Normal SMD
Low SMD
60
0
40
20
Time to treatment (months)

## Slide 6
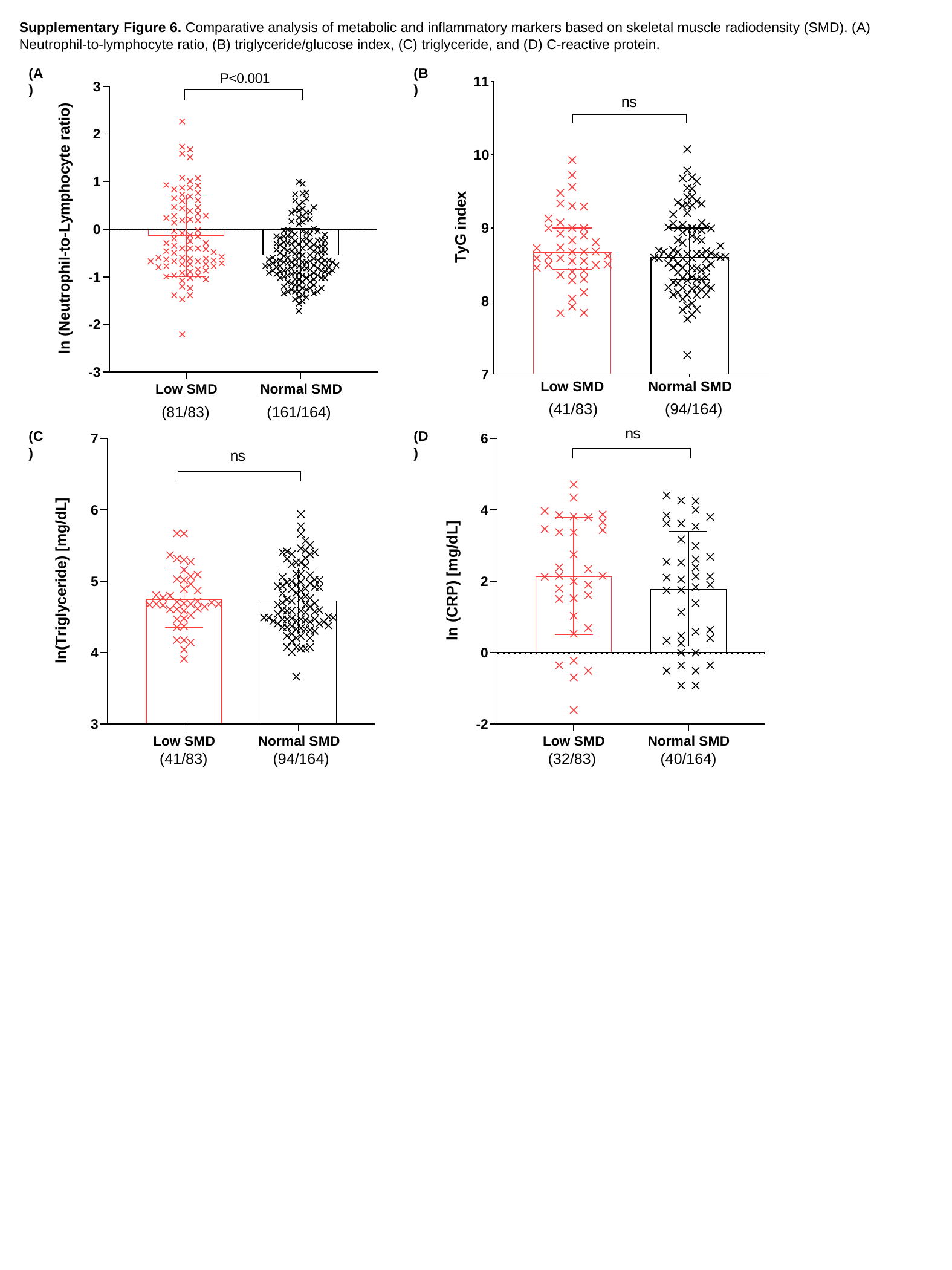

Supplementary Figure 6. Comparative analysis of metabolic and inflammatory markers based on skeletal muscle radiodensity (SMD). (A) Neutrophil-to-lymphocyte ratio, (B) triglyceride/glucose index, (C) triglyceride, and (D) C-reactive protein.
(A)
(B)
(C)
(D)
