# Supplementary material for: Low Skeletal Muscle Radiodensity Predicts Response to CDK4/6 Inhibitors Plus Aromatase Inhibitors in Advanced Breast Cancer
Source: J Cachexia Sarcopenia Muscle. 2024 Dec 17;16(1):e13666. doi: 10.1002/jcsm.13666 (PMC11670169; doi:10.1002/jcsm.13666)
Supplement: Supplementary file 3 — Table S1. Univariate and multivariate analyses of factors associated with progression‐free survival categorized by body composition parameters (lower tertile group). Table S2. Hazard ratio (HR) and p value summary for progression‐free survival (PFS) and overall survival (OS) between normal skeletal muscle radiodensity (SMD) and low SMD in premenopausal, postmenopausal, with VM, and without VM subgroups. Table S3. Body composition parameters adjusted for age and body mass index (BMI) in relation to grade 3 or higher neutropenia. Table S4. Number and percentage of patients classified as having sarcopenia based on different skeletal muscle index (SMI) cut‐off values. [file JCSM-16-e13666-s003.docx]

**Supplementary Table 1.** Univariate and multivariate analyses of factors associated with progression-free survival categorized by body composition parameters (lower tertile group).

|  |  | | **Univariable** |  | |  | **Multivariable** |  | |  |
| --- | --- | --- | --- | --- | --- | --- | --- | --- | --- | --- |
| **Variable** |  | | **HR (95% CI)** | **P-value** | |  | **HR (95% CI)** | **P-value** | |  |
| **Age**, by 5 years | |  | 1.01 (0.93 – 1.08) | | 0.89 |  | 0.96 (0.88 – 1.04) | | 0.27 | |
| **Visceral metastasis** | | Yes | 1.59 (1.08 – 2.32) | | 0.017^†^ |  | 1.63 (1.12 – 2.39) | | 0.012^†^ | |
| **L3–SMI** | | Mid to upper tertiles | Reference | |  |  |  | |  | |
|  | | Lowest tertile | 1.03 (0.69 – 1.52) | | 0.90 |  |  | |  | |
| **L3–AVFI** | | Mid to upper tertiles | Reference | |  |  |  | |  | |
|  | | Lowest tertile | 0.96 (0.65 – 1.42) | | 0.82 |  |  | |  | |
| **L3–SFI** | | Mid to upper tertiles | Reference | |  |  |  | |  | |
|  | | Lowest tertile | 0.84 (0.57 – 1.23) | | 0.37 |  |  | |  | |
| **L3–SMD** | | Mid to upper tertiles | Reference | |  |  | Reference | |  | |
|  | | Lowest tertile | 1.63 (1.13 – 2.36) | | 0.009^†^ |  | 1.84 (1.22 – 2.76) | | 0.003^†^ | |

AVFI, Abdominal visceral fat index; BMI, Body mass index; DM, Diabetes mellitus; L3, third lumbar spine vertebra; SFI, Subcutaneous fat index; SMD, Skeletal muscle radiodensity; SMI, Skeletal muscle index.
^†^ P-value < 0.05

**Supplementary Table 2.** Hazard ratio (HR) and p value summary for progression-free survival (PFS) and overall survival (OS) between normal skeletal muscle radiodensity (SMD) and low SMD in premenopausal, postmenopausal, with VM, and without VM subgroups.

|  | PFS | |  | OS | |
| --- | --- | --- | --- | --- | --- |
|  | HR^†^ (95% CI) | P-value |  | HR^†^ (95% CI) | P-value |
| Premenopausal | 3.04 (1.54-5.99) | 0.001 |  | 3.57 (1.45-8.76) | 0.006 |
| Postmenopausal | 1.19 (0.76-1.87) | 0.443 |  | 2.27 (1.23-4.20) | 0.009 |
| Without VM | 2.94 (1.58-5.45) | <0.001 |  | 5.33 (2.13-13.34) | <0.001 |
| With VM | 1.17 (0.71-1.90) | 0.539 |  | 1.79 (0.95-3.38) | 0.071 |

CI, Confidence interval; HR, Hazard ratio; OS, Overall survival; PFS, Progression free survival, SMD, Skeletal muscle radiodensity; VM, Visecral metastasis.

^†^ HR was calculated using normal SMD group as the reference.

**Supplementary Table 3.** Body composition parameters adjusted for age and body mass index (BMI) in relation to grade 3 or higher neutropenia.

|  |  | **Univariable** |  | |  |  | **Multivariable^‡^** |  |
| --- | --- | --- | --- | --- | --- | --- | --- | --- |
| **Variable** |  | **OR (95% CI)** | | **P-value** |  |  | **OR (95% CI)** | **P-value** |
| **Age**, by 5 years | | 0.97 (0.87 – 1.07) | | 0.52 |  |  |  |  |
| **Baseline BMI** | ≥ 25 | 0.92 (0.51 – 1.66) | | 0.77 |  |  |  |  |
| **L3–SMI** | Upper tertile | 0.91 (0.53 – 1.56) | | 0.74 |  |  | 0.88**^‡^** (0.48 – 1.59) | 0.71 |
| **L3–AVFI** | Upper tertile | 0.56 (0.33 – 0.95) | | 0.03^†^ |  |  | 0.49**^‡^** (0.26 – 0.94) | 0.03^†^ |
| **L3–SFI** | Upper tertile | 1.18 (0.69 – 2.04) | | 0.55 |  |  | 1.30**^‡^** (0.69 – 2.47) | 0.41 |
| **L3–SMD** | Upper tertile | 1.13 (0.66 – 1.94) | | 0.66 |  |  | 1.06**^‡^** (0.59 – 1.91) | 0.84 |

AVFI, Abdominal visceral fat index; BMI, Body mass index; DM, Diabetes mellitus; L3, third lumbar spine vertebra; SFI, Subcutaneous fat index; SMD, Skeletal muscle radiodensity; SMI, Skeletal muscle index.
^†^ P-value < 0.05

^‡^ The OR of each body composition parameter was adjusted for age and BMI.

**Supplementary Table 4.** Number and percentage of patients classified as having sarcopenia based on different skeletal muscle index (SMI) cut-off values.

| **SMI cut-off value** | **Patients with Sarcopenia, No. (%)** | **P-value**^†^ |
| --- | --- | --- |
| Prado et al. Lancet Oncol. 2008  (SMI <38.5 cm^2^/m^2^ for women) | 93 (37.7) | <0.001 |
| Fearon et al. Lancet Oncol. 2011*  (SMI <39 cm^2^/m^2^ for women) | 104 (42.1) |  |
| Martin et al. J Clin Oncol. 2013  (SMI <41 cm^2^/m^2^ for women) | 136 (55.1) |  |
| -1SD below  (SMI <34.4 cm^2^/m^2^ for women) | 40 (16.2) |  |
| -1SD below, based on BMI categories:  (SMI <33.7 cm²/m² for women with BMI <25,  SMI < 39.7 cm²/m² for women with BMI ≥ 25) | 37 (15.0) |  |

BMI, Body mass index; No; Number; SD, Standard deviation; SMI, skeletal muscle index.

* The international definition

^†^ P-value was calculated using Cochran’s Q test.
